# Supplementary material for: Correlating metasurface spectra with a generation-elimination framework
Source: Nat Commun. 2023 Aug 12;14:4872. doi: 10.1038/s41467-023-40619-w (PMC10423275; doi:10.1038/s41467-023-40619-w)
Supplement: Supplementary file 1 — Supplementary Information [file 41467_2023_40619_MOESM1_ESM.pdf]

Supplementary Information for

Correlating metasurface spectra with a generation-elimination framework

Jieting Chen[1,2,3], Chao Qian[1,2,3,*], Jie Zhang[1,2,3,], Yuetian Jia[1,2,3], and Hongsheng Chen[1,2,3,*]

[1] *ZJU-UIUC Institute, Interdisciplinary Center for Quantum Information, State Key Laboratory of Extreme Photonics and Instrumentation, Zhejiang University, Hangzhou 310027, China.*

[2] *ZJU-Hangzhou Global Science and Technology Innovation Center, Key Lab. of Advanced Micro/Nano Electronic Devices & Smart Systems of Zhejiang, Zhejiang University, Hangzhou 310027, China.*

[3] *Jinhua Institute of Zhejiang University, Zhejiang University, Jinhua 321099, China.*

[*] *Corresponding authors: chaoq@intl.zju.edu.cn (C. Qian); hansomchen@zju.edu.cn (H. Chen)*

This PDF file includes:

- Supplementary Note 1: Mathematical illustration and loss objective
- Supplementary Note 2: Details of the network architecture
- Supplementary Note 3: Visualizing Gaussian distributions in latent space
- Supplementary Note 4: Supervised learning loss of generation and elimination network
- Supplementary Note 5: Additional results on other geometrical patterns
- Supplementary Note 6: Pattern-generating procedure with the relevant algorithms

Supplementary Note 1: Mathematical illustration and loss objective

As indicated in Supplementary Fig. 1, there are three types of variables in our VAE-based sub-networks: input or output variable x, label variable y, and latent variable z. To be more precise, our sub-networks are actually CVAE-based [S1] and can be viewed as one type of VAE [S2,S3] that performs auto-encoding of the input or output variable x conditioned on the label y. The whole training phase can be decomposed into conditional recognition process and generative process, corresponding to the encoder and decoder, respectively. Note that, in the inference procedure, only the generative process is carried out. In other words, CVAE operates $q_{\varphi}(z|x,y)$ during training, but it uses $p_{\theta}(z|y)$ to draw samples of z and make diverse output predictions during the generative process.

The mathematical conditional generative process of the sub-network is depicted in Supplementary Fig. 1c. For a given label observation y, the latent variable z is drawn from the prior distribution $p_{\theta}(z|y)$, and the output x is generated from the distribution $p_{\theta}(x|z,y)$. Compared with conventional fully-connected network (Supplementary Fig. 1a), the latent variable z allows for modeling multiple modes in the conditional distribution of output variables x given label observation y, making the proposed generation or elimination sub-network suitable for modeling one-to-many mapping.

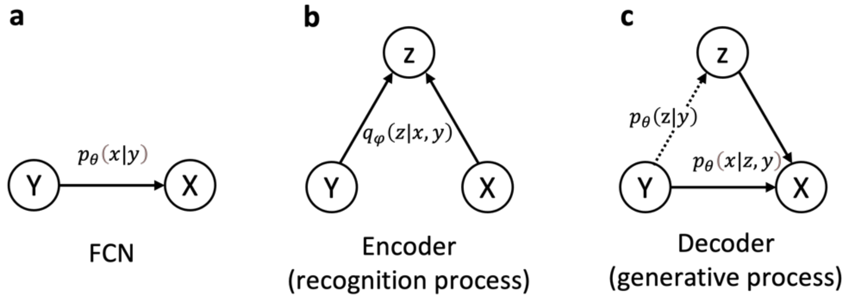

Supplementary Figure 1 | Graphic mathematical relationship of three variables. a, A direct inference from y to x is adopted in conventional fully-connected network. **b**, An approximate inference of latent variable z, also known as encoder or recognition process of sub-networks. **c**, The decoder or generative process of sub-networks. The dashed line means z is directly sampled from $\mathcal{N}(0,I)$ during the inference phase with the testing or user-defined label y.

Here we utilize a trick called variational inference approximation to match the true posterior with approximate posterior distribution $q_\varphi(z|x,y)$, where the variational parameter φ indexes the family of distributions. As illustrated in the main text, we define a Kullback-Leibler divergence term $KL[q_\varphi(z|x,y)||p_\theta(z|x,y)]$ to evaluate the information loss. Based on the Bayes rule, this loss function can be rewritten as

$$KL[q_\varphi(z|x,y)||p_\theta(z|x,y)] = \mathbb{E}_{q_\varphi(z|x,y)}[log\, q_\varphi(z|x,y) - log\, p_\theta(z|x,y)] \tag{S1}$$

$$= \mathbb{E}_{q_\varphi(z|x,y)}[log\, q_\varphi(z|x,y) - log \frac{p_\theta(x,z|y)}{p_\theta(x|y)}] \tag{S2}$$

$$= \mathbb{E}_{q_\varphi(z|x,y)}[log\, q_\varphi(z|x,y) - log\, p_\theta(x,z|y)] + log\, p_\theta(x|y) \tag{S3}$$

The ultimate goal is to seek out the optimal variational parameter φ that minimizes the above loss function. Under our Gaussian hypothesis, φ would be the mean and variance of the latent variables for each data point x_i, i.e., $\varphi_{x_i} = (\mu_{x_i}, \sigma_{x_i}^2)$. We choose Gaussian as the prior distribution because of its analytical evaluation of the variation loss (see Eq. S9-S11), and the convenience of performing sampling in the latent space.

In general, the pesky evidence term $log\, p_\theta(x|y)$ in Eq. (S3) is impossible to be computed directly, as it requires exponential time to integrate $log\, p_\theta(x|y) = \int p_\theta(x|z,y)\, p_\theta(z|y)\, dz$ over all configurations of latent variables. To tackle the challenge, we define a new term:

$$ELBO(\varphi) = \mathbb{E}_{q_\varphi(z|x,y)}[log\, p_\theta(x,z|y) - log\, q_\varphi(z|x,y)] \tag{S4}$$

and Eq. (S3) is transformed into

$$log\, p_\theta(x|y) = ELBO(\varphi) + KL[q_\varphi(z|x,y)||p_\theta(z|x,y)] \tag{S5}$$

The network is trained to maximize the conditional log-likelihood in LHS of Eq. (S5). According to Jensen's inequality, the Kullback-Leibler divergence is always greater than or equal to zero, that is, minimizing the KL divergence term in Eq. (S5) is equivalent to maximizing the ELBO (the evidence lower bound, also known as variational lower bound). Now we are saved from having to calculate

and minimize the KL divergence between the approximate and the true posteriors. Instead, our training objective is converted into maximizing ELBO, and we can rewrite Eq. (S5) as

$$\log p_\theta(x|y) \geq ELBO(\varphi)$$

$$= \mathbb{E}_{q_\varphi(z|x,y)}[\log p_\theta(x|z,y) + \log p_\theta(z|y) - \log q_\varphi(z|x,y)] \tag{S6}$$

$$= \mathbb{E}_{q_\varphi(z|x,y)}[\log p_\theta(x|z,y)] - KL[q_\varphi(z|x,y)||\, p_\theta(z|y)] \tag{S7}$$

Therefore, a surrogate objective loss function is defined as negative of $ELBO(\varphi)$:

$$\mathcal{L}_{CVAE}(x,y;\theta,\varphi) = KL[q_\varphi(z|x,y)||\, p_\theta(z|y)] - \mathbb{E}_{q_\varphi(z|x,y)}[\log p_\theta(x|z,y)] \tag{S8}$$

where the first RHS term is KL divergence loss, and the second term is an expected reconstruction loss. During the optimization, the reconstruction loss and KL divergence loss are always in opposition to each other until convergence. The KL divergence term can be interpreted as regularizing the Gaussian parameter φ, encouraging the approximate posterior $q_\varphi(z|x,y)$ to be as close to the prior $p_\theta(z)\sim\mathcal{N}(0,I)$ as possible. This term equips the model with generalization capability to generate diverse candidates. If we delete it, the model will degenerate into the autoencoder that is designed for one-to-one mapping.

To further simplify Eq. (S8), the divergence term $KL[q_\varphi(z|x,y)||\, p_\theta(z|y)]$ can be integrated analytically, while the expectation term requires the estimation by Monte Carlo sampling and the reparameterization trick. Note that, though the prior of the latent variable is modulated by the label y in our formulation, the constraint can be easily relaxed, i.e., $p_\theta(z|y) = p_\theta(z)$ [S2]. Due to $p_\theta(z)\sim\mathcal{N}(0,I)$ and $q_\varphi(z|x,y)\sim\mathcal{N}(\mu,\sigma)$, the KL divergence term in Eq. (S8) can be simplified as follows:

$$KL[q_\varphi(z|x,y)||\, p_\theta(z|y)] = KL[\mathcal{N}(\mu,\sigma^2)||\mathcal{N}(0,I)] \tag{S9}$$

$$= \int \frac{1}{\sqrt{2\pi\sigma^2}} e^{-\frac{(x-\mu)^2}{2\sigma^2}} \left(\log \frac{\frac{1}{\sqrt{2\pi\sigma^2}} e^{-\frac{(x-\mu)^2}{2\sigma^2}}}{\frac{1}{\sqrt{2\pi}} e^{-x^2/2}}\right) dx \tag{S10}$$

$$= \frac{1}{2}(\mu^2 + \sigma^2 - \log\sigma^2 - 1) \tag{S11}$$

To estimate the expectation term, a straightforward approach is to draw samples z from the posterior probability and take the average of the likelihoods. We call this method the Monte Carlo sampling, and the expectation term in Eq. (S8) is induced into

$$\mathbb{E}_{q_{\varphi}(z|x,y)}[log\, p_{\theta}(x|z,y)] \simeq \frac{1}{L}\sum_{l=1}^{L}\left[log\, p_{\theta}\left(x|z^{(l)},y\right)\right],\ z^{(l)}\sim q_{\varphi}(z|x,y) \tag{S12}$$

Furthermore, a reparameterization trick [S3] is applied to make the evidence lower bound (i.e., variational lower bound) differentiable. Assisted with an (auxiliary) noise variable ϵ, the loss function is finally simplified into

$$\mathcal{L}_{CVAE}(x,y;\theta,\varphi) = \frac{1}{2}(\mu^2+\sigma^2-log\sigma^2-1)-\frac{1}{L}\sum_{l=1}^{L}\left[log\, p_{\theta}\left(x|z^{(l)},y\right)\right],$$

$$z^{(l)} = g_{\varphi}(x,\varepsilon^{(l)}),\ \varepsilon^{(l)}\sim p(\epsilon)$$

$$\text{where}\qquad z^{(l)} = \mu+\sigma\odot\varepsilon^{(l)},\ \varepsilon^{(l)}\sim\mathcal{N}(0,I) \tag{S14}$$

The above process which further simplifies Eq. (S8) is called Stochastic Gradient Variational Bayes (SGVB) estimation [S3].

Using variational lower bound (ELBO) as the loss objective and the reparameterization trick, our model can be easily trained via stochastic optimization. Once the model parameters are learned, we can make a prediction of an output x from a label y by following the reconstruction module of the network, i.e., $x' = argmax_x\, p_{\theta}(x|\, z',y)$, $z' = \mathbb{E}[z|y]$. In our implementation, z' is randomly sampled from $\mathcal{N}(0,I)$, as indicated by the dashed line in Supplementary Fig. 1c. In theory, we should sample z from the variational posterior $q_{\varphi}(z|x,y)$. However, minimizing the KL divergence term in Eq. (S8) indicates that the parameters (μ and σ) of $q_{\varphi}(z|x,y)$ are optimized to closely resemble that of the prior probability $p_{\theta}(z)\sim\mathcal{N}(0,I)$ [S2], making it reasonable to sample directly from the standard Gaussian distributions.

Supplementary Note 2: Details of the network architecture

Autoencoder architecture. To visualize the data distributions in latent spaces, we use two autoencoders to perform dimensionality reduction on continuous spectra data, degrading the nonrepresentational data into one-dimensional encodings e_1 (40~60 THz) and e_2 (60~100 THz).

The autoencoder is mainly composed of two parts: an encoder and a decoder. As shown in Supplementary Fig. 2, three input reflection coefficients, R_{xx}, R_{xy} and R_{yy}, are encoded separately before concatenation, and further encoded into a 5-dimensional vector. The decoding is a mirrored symmetrically process, where a 30-dimensional vector is split into three 10-dimensional vectors and decoded severally; see detailed operations and layer parameters in Supplementary Table 1. When the autoencoder is trained to be capable of reconstructing input spectra, the 5-dimensional vector in the mid-layer of the network will be taken out and used as feature representation, where a summation operation is further adopted for ultimate one-dimensional encoding.

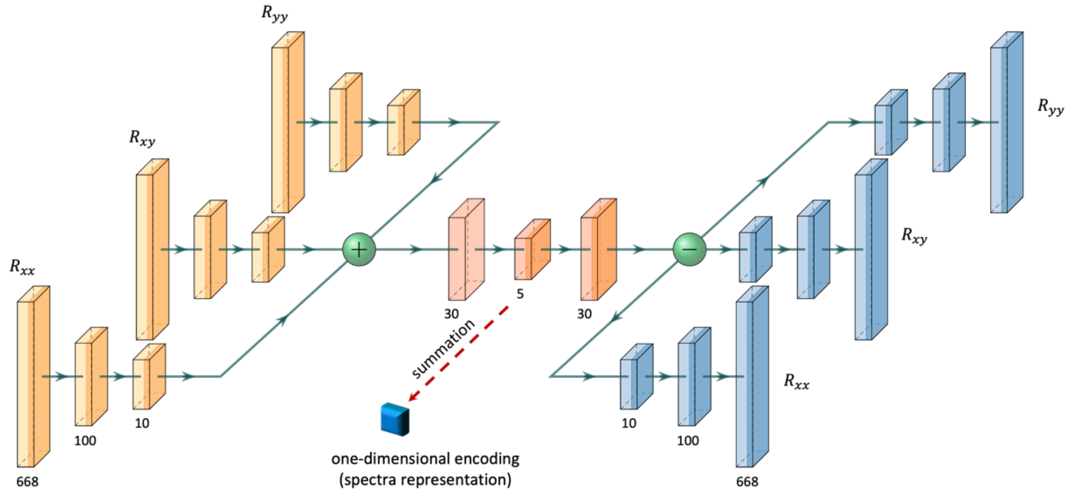

Supplementary Figure 2 | Schematic of autoencoder architecture. An innovative and effective approach to compress the abstract high-dimensional data into continuous one-dimensional data. Three reflection coefficients, R_{xx}, R_{xy} and R_{yy}, are encoded separately before concatenation and decoded severally after splitting. After the autoencoder is trained to be capable of reconstructing input spectra, an additional summation operation is performed to finally obtain one-dimensional spectra representation.

layer		op.	size-in	size-out	
1 (R_{xx}, R_{xy}, R_{yy})		-	668	-	
2 (R_{xx}, R_{xy}, R_{yy})		fc<br>relu	668	100	
3 (R_{xx}, R_{xy}, R_{yy})		fc<br>linear	100	10	Encoder
4		concatenation	10×3	30	
5		fc<br>linear	30	5	
6		fc<br>linear	5	30	
7 (R_{xx}, R_{xy}, R_{yy})		split	30	10×3	Decoder
8 (R_{xx}, R_{xy}, R_{yy})		fc<br>relu	10	100	
9 (R_{xx}, R_{xy}, R_{yy})		fc<br>relu	100	668	

Supplementary Table 1 | Definition and parameters of the autoencoder. The encoder consists of the first five layers, while the decoder is composed of the last five layers. "fc" refers to the fully-connected layer; "relu" refers to the ReLU activation function; "linear" refers to the linear activation function.

Generation network architecture. The architecture of the generation network that is constituted by an encoder, a latent space, and a decoder is schematically depicted in Fig. 2 with detailed parameters listed in Supplementary Table 2. The feature extraction module composed of 4 fully-connected layers compresses input spectra into lower dimensions. The reconstruction module is composed of 5 transposed fully-connected layers (the last layer, named "Output", is an additional "T_FC"). The "Label" in the 6th and 13th layers refers to the low-frequency spectrum y_i for each data point x_i.

	layer	name	op.	size-in	size-out	
	1	Input	-	2004	-	Encoder
Feature extraction module	2	FC1	fc relu	2004	1024	Encoder
Feature extraction module	3	FC2	fc relu	1024	512	Encoder
Feature extraction module	4	FC3	fc relu	512	512	Encoder
Feature extraction module	5	FC4	fc relu	512	256	Encoder
	6	Label	-	999	-	Encoder
	7	Concat1	concatenation	256+999	1255	Encoder
	8	Concat2	fc relu	1255	512	Encoder
	9	Concat3	fc relu	512	256	Encoder
	10	mu	fc linear	256	2	Latent space
	11	sigma	fc linear	256	2	Latent space
	12	Sampled	sampling	2	2	Latent space
	13	Label	-	999	-	Decoder
	14	Concat4	concatenation	2+999	1001	Decoder
Reconstruction module	15	T_FC1	fc relu	1001	256	Decoder
Reconstruction module	16	T_FC2	fc relu	256	512	Decoder
Reconstruction module	17	T_FC3	fc relu	512	512	Decoder
Reconstruction module	18	T_FC4	fc relu	512	1024	Decoder
Reconstruction module	19	Output	fc sigmoid	1024	2004	Decoder

Supplementary Table 2 | Definition and parameters of the generation network architecture. The feature extraction module combined with *Concat1-3* composes the encoder, while the reconstruction module combined with *Concat4* composes the decoder. Latent space includes fully-connected operations for mean (“mu”) and standard deviation units (“$sigma$”) followed by Gaussian sampling (“Sampled”). “T_FC” refers to the transposed fully-connected layer; “sigmoid” refers to the sigmoid activation function.

Baseline fully-connected network (FCN) architecture. The detailed architecture of the FCN used for comparison is shown in Supplementary Table 3. The baseline model is composed of 9 fully-connected layers with 300 hidden neurons in each layer. Correspondingly, the training (orange line) and validation (blue line) loss curves are plotted in Supplementary Fig. 3. The relatively large fluctuations in the loss curves indicate the failure of convergence. Besides, the validation error stays at a high level (around $4e^{-3}$ measured in MSE), compared to $5e^{-4}$ in our proposed framework. The error of nearly one order of magnitude not only proves that the FCN fails to converge, but also exposes its performance gap with our framework when confronted with the same bidirectional non-uniqueness

predicament. We further increase the number of layers to 10 and 11 as larger models; however, the non-convergence problem is not eased.

In addition, we try to use three independent FCN networks to train Rxx, Rxy and Ryy, separately. Supplementary Table 4 displays the numerical results of utilizing the three criteria to evaluate the performance of baseline FCN, three FCNs and our framework. The close and relatively low values of baseline FCN and three FCNs indicate that the strategy of using three networks to represent Rxx, Rxy, Ryy, separately, does not alleviate the spectral correlation mapping issue. Besides, the quantitative differences between three FCNs and our framework indicate that the performance gap still exists.

layer		name	op.	size-in	size-out
1		Input	-	2004	-
2		dense1	fc<br>relu	2004	300
3		dense2	fc<br>relu	300	300
4		dense3	fc<br>relu	300	300
5		dense4	fc<br>relu	300	300
6		dense5	fc<br>relu	300	300
7		dense6	fc<br>relu	300	300
8		dense7	fc<br>relu	300	300
9		dense8	fc<br>relu	300	300
10		prediction	fc<br>linear	300	999

Supplementary Table 3 | Definition and parameters of the baseline model FCN used for comparison. The baseline FCN model is composed of 9 fully-connected layers with 300 hidden neurons in each layer.

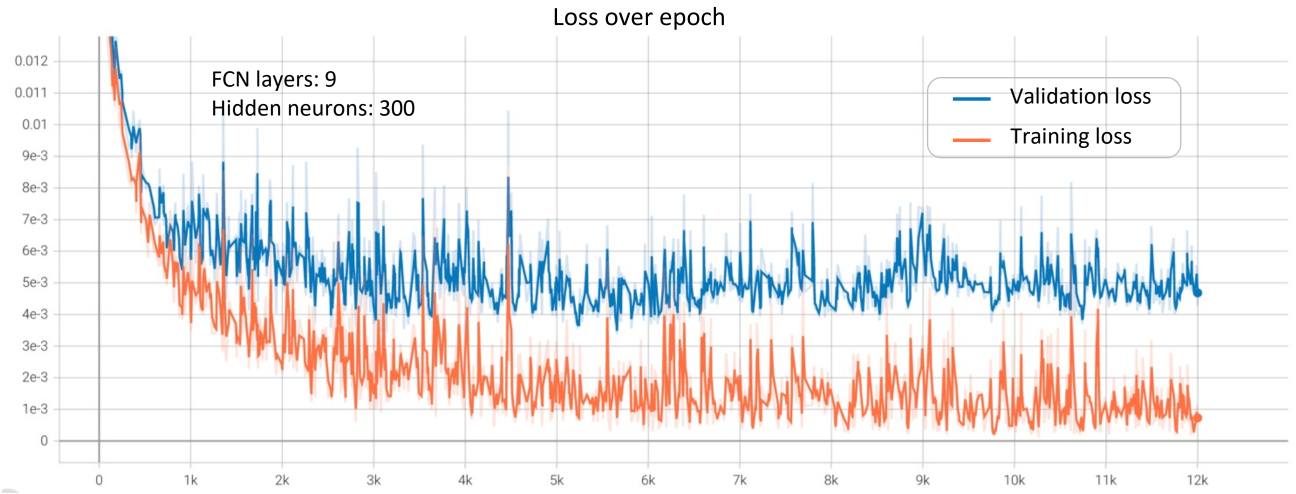

Supplementary Figure 3 | The training and validation loss of FCN measured in MSE. The blue line is the validation loss and the orange line is the training loss. Both curves have been smoothed with a smoothing factor = 0.4. The validation error fluctuates vigorously above 4e^{-3}.

	MSE	Average acc.	Similarity		
			R_{xx}	R_{xy}	R_{yy}
Our framework	5.174e-4	98.77%	98.13%	96.38%	98.15%
Baseline FCN	4.015e-3	95.25%	86.96%	86.68%	82.42%
Three FCNs	3.858e-3	93.50%	84.37%	86.21%	84.94%

Supplementary Table 4 | The quantitative comparisons between baseline FCN, three FCNs and our framework. In three FCNs model, three independent FCN networks are used to train Rxx, Rxy and Ryy, separately, each with the same configuration as the baseline model in Supplementary Table 3 except for the input size and output size.

Supplementary Note 3: Visualizing Gaussian distributions in latent space

As elucidated in the main text, Fig. 3a shows the two-dimensional (2D) distribution of 600 encoded training data featured or colored by 40~60 THz spectra representation e_1. Whenever it is assigned with a small range of values, a Gaussian distribution will be extracted, as shown in Supplementary Fig. 4. The distributions are not perfectly standard Gaussian, otherwise the KL divergence loss term in Eq. (S1) would be zero, leading to a big reconstruction loss with unrealistic reconstructed results. Nevertheless, when two losses in counterbalance reach the convergence, the true latent distribution

conditioned on any e_1 would be close to standard Gaussian distribution, making it reasonable to sample latent variables directly from the prior probability $p_\theta(z) \sim \mathcal{N}(0,1)$.

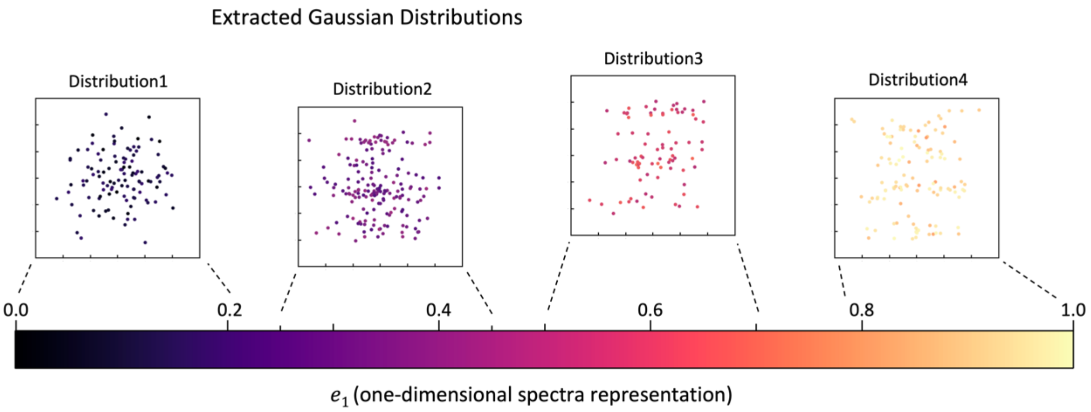

Supplementary Figure 4 | Extracted Gaussian distributions. Whenever e_1 (input spectra representation on 40~60 THz) is assigned with a small range of values, a Gaussian distribution will be extracted. In each distribution, all points are nominated high-frequency spectra from the training dataset, corresponding to the low-frequency spectra that e_1 stands for.

Supplementary Note 4: Supervised learning loss of generation and elimination network

We firstly trained the generation network on 100,000 epochs with its training loss and validation loss delineated in right of Supplementary Fig. 5a. The increasing gap between the training loss and the validation loss starting from 20,000 epochs indicates the portent of overfitting. Therefore, we cut off the training epochs at 10,000 for the generation network, as an early stopping regularization measure [S4]. Similarly, for the elimination network, a slight overfitting phenomenon occurs in the first 10,000 epochs (Supplementary Fig. 5b). We cut off the training epochs at 4,000 without compromising on model accuracy.

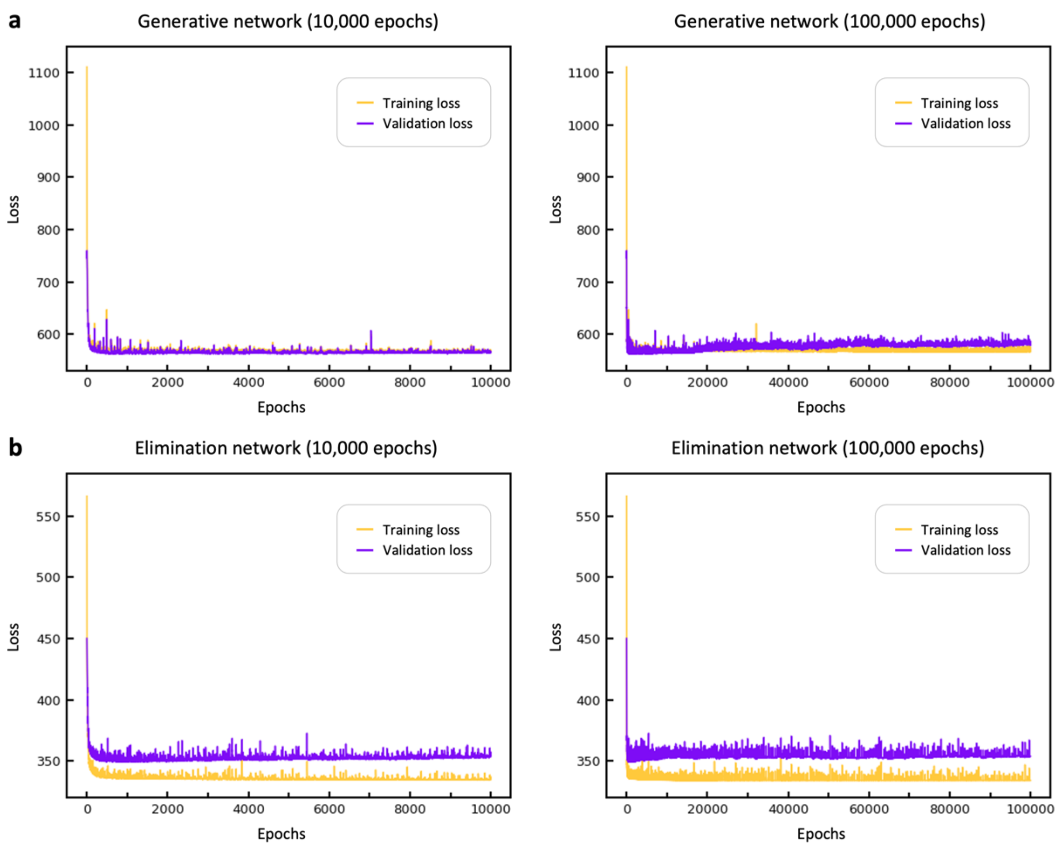

Supplementary Figure 5 | The loss of generation network and elimination network over epochs. a, The training and validation losses on generation network over 10,000 epochs and 100,000 epochs. The increasing gap between the training loss and the validation loss starting from 20,000 epochs indicates the portent of overfitting. **b,** The training and validation losses on elimination network over 10,000 epochs and 100,000 epochs. A slight overfitting phenomenon occurs in the first 10,000 epochs.

Supplementary Note 5: Additional results on other geometrical patterns

Apart from the representative elliptical-shaped metasurface that is demonstrated in the main text, our model has a strong generalization ability which can be extended to other possible geometrical patterns. In Supplementary Figs. 6 and 7, we show the intuitional results of both sub-networks after being fine-tuned on two additional datasets. The datasets are obtained by running numerical simulations on arc and distorted h-shape metasurface patterns, respectively. Even though the degree of freedom is increased (at least eight for the distorted h-shape pattern) for both cases, the dimension of the latent space is still set as two and can achieve relatively high accuracy. The first plot in each row is the input spectra from the testing dataset, and the second plot is the diverse candidates generated by the generation network. The last plot in each row is the final solution singled out by the

elimination network, where the solid line and inset are the ground-truth optical response and design pattern, respectively. The close match between the dashed line and solid line in all cases proves the generality of our model. Furthermore, Supplementary Table 5 displays the quantified results of three geometries measured by the criteria that defined in the main text. Whatever the geometry type is, the MSE errors are quite low, the average accuracy and the similarity are relatively high. All of these guarantee the versatility of the proposed framework and exemplify that the type of structure does not affect the accuracy of our model.

Structure	MSE	Average acc.	Similarity		
			R_{xx}	R_{xy}	R_{yy}
Ellipse	5.174e-4	98.77%	98.13%	96.38%	98.15%
Arc	1.066e-3	97.85%	95.24%	96.84%	96.20%
Distorted h-shape	3.516e-4	98.57%	96.14%	98.56%	95.84%

Supplementary Table 5 | The quantified evaluation of the framework on various structures.

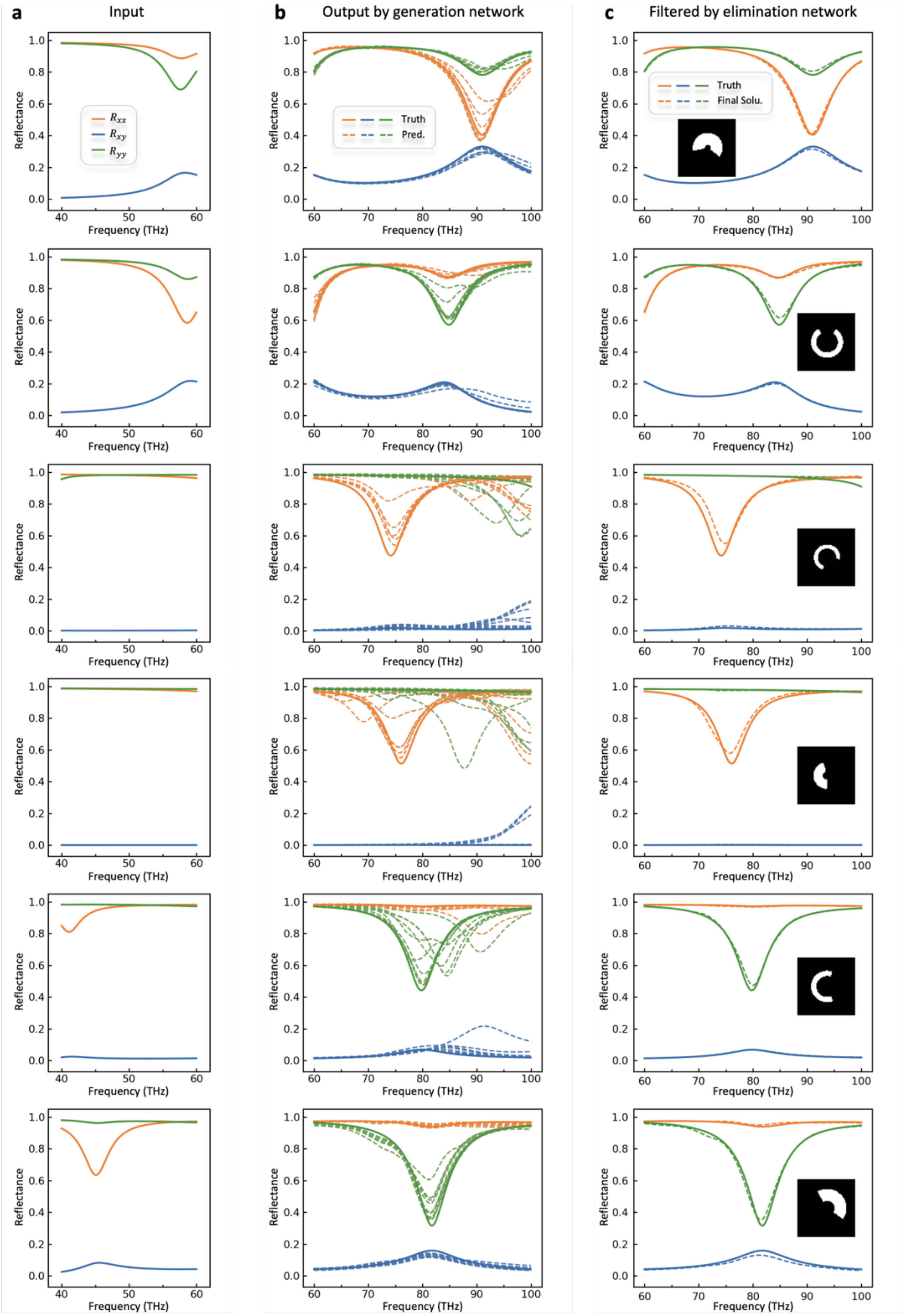

Supplementary Figure 6 | Evaluation of the generation-elimination framework on arc dataset. a, The input spectra of six randomly-selected samples from the arc testing dataset, where three

reflection coefficients, R_{xx}, R_{xy} and R_{yy} are plotted for each sample. **b**, The results outputted by the generation network. At least one of the candidates (the dashed lines) is consistent with the ground truth (the solid line). **c,** Final solutions singled out by the elimination network. The solid lines and insets are the ground-truth reflection spectra and their metasurface designs, respectively. The dashed lines are filtering results of the elimination network.

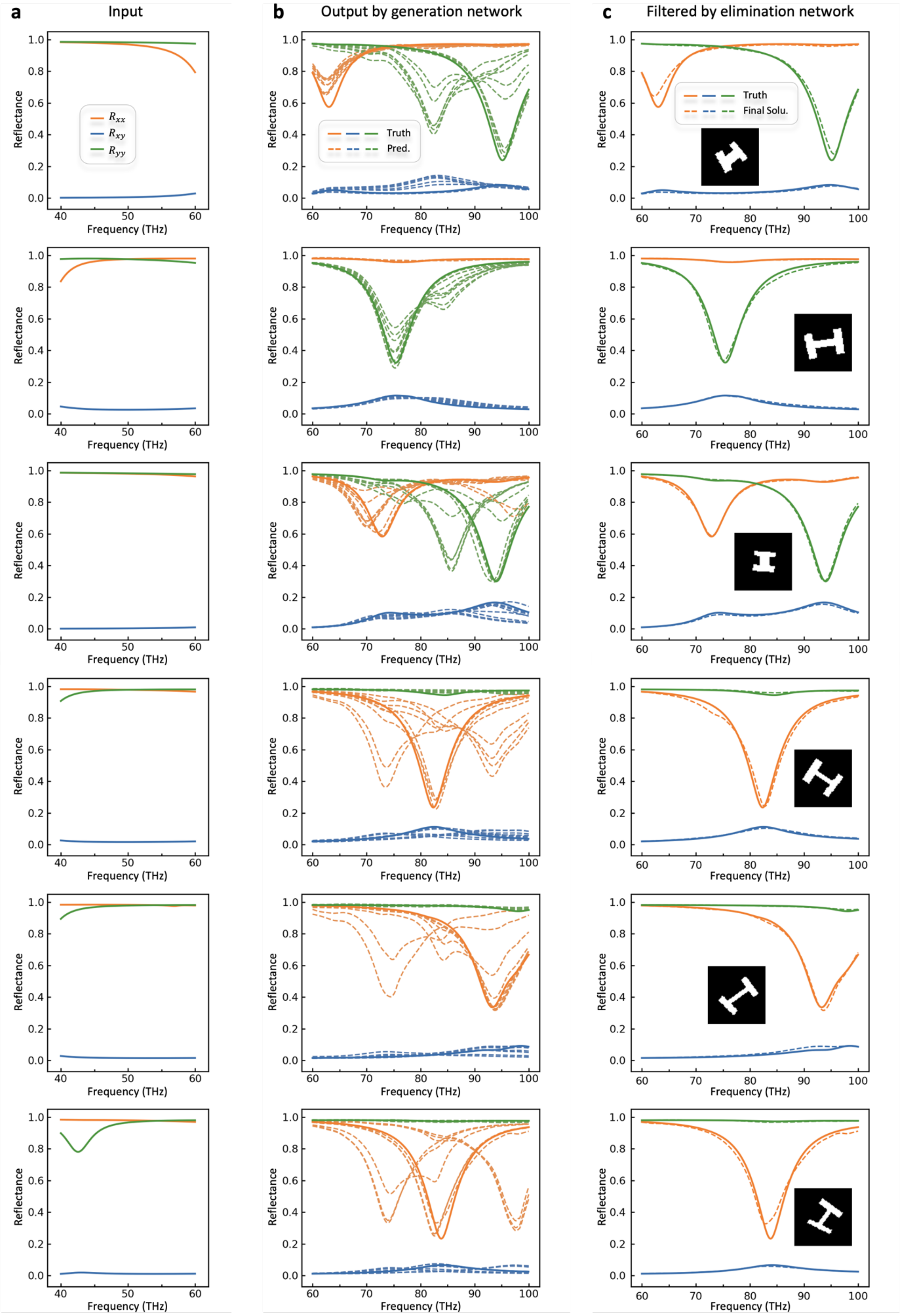

Supplementary Figure 7 | Evaluation of the generation-elimination framework on distorted h-

shape dataset. The six samples are randomly selected from the distorted h-shape testing dataset. Other settings are similar to Supplementary Fig. 5.

Supplementary Note 6: Pattern-generating procedure with the relevant algorithms

As elucidated in the Methods in the main text, various geometry types of metasurface patterns are created in Python and run simulation using the MATLAB-CST co-simulation method. The detailed procedure is depicted in Supplementary Fig. 8 and can be listed as three steps.

Step 1: Create the binary black/white images in Python by sampling over all possible design parameters, such as length, width, diameter and rotation angle of different parts of the geometries. Three relevant pattern-generating algorithms that written in pseudo-code are appended below; the main built-in packages (including rotation and distortion) and invoked functions are underlined for clarity.

Step 2: Then, these binary images are exported to individual .txt files as 64 × 64 matrixes (i.e., one matrix in a file), which are prepared to be taken as inputs in the next step using the MATLAB-CST co-simulation method.

Step 3: In the numerical simulation, MATLAB reads these binary matrixes from.txt files and constructs them in CST, where '1' stands for gold and '0' stands for air. Other parameters such as periodicity and thickness are also pre-defined in MATLAB, to control the modeling in CST and then numerically calculate the spectra.

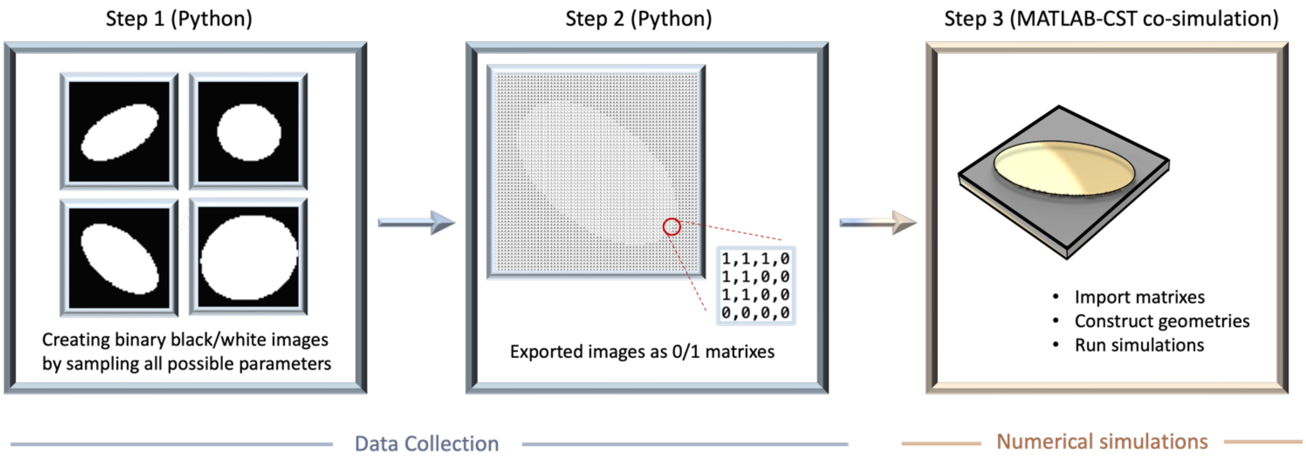

Supplementary Figure 8 | Illustrative procedure of constructing the ellipse geometry. The three steps to construct elliptical-shaped metasurface patterns in CST and obtain the reflection spectra (the

same procedure for the other geometries). The first two steps are executed in Python and the third step is performed by MATLAB-CST co-simulation.

Algorithm 1 Ellipse Pattern Generation

```
for axesLength_X = 2, 4, ..., 28, 30 do
    for axesLength_Y = 2, 4, ..., 28, 30 do
        for angle = 0, 10, ..., 80 do
            Initialize img ← 64 × 64 zeros
            img = cv2.ellipse(img, axesLength_X, axesLength_Y, angle)
            Export and save img as 64 × 64 binary matrix
            if axesLength_X == axesLength_Y (degrade as circle) then
                Break
            end if
        end for
    end for
end for
```

Algorithm 2 Arc Pattern Generation

```
for diameter, start_angle, total_angle, linewidth do
▷ all parameters are adequately and randomly sampled within a reasonable range
    width, height ← diameter
▷ here we only exhibit the main parameters when calling functions
    img = matplotlib.pyplot.Arc(center, width, height, start_angle, theta1 = 0, theta2 = total_angle, linewidth)
    img = cv2.resize(img, dsize = (64, 64))
    Transform img into binary image
    Export and save img as 64 × 64 binary matrix
▷ for Sector Pattern Generation, invoke matplotlib.pyplot.Wedge in the same way
end for
```

Algorithm 3 Distorted H-shape Pattern Generation

```
function H_SHAPE_DRAWING(main_comb, side_comb1, side_comb2, angle, add_angle)
    Initialize img ← 64 × 64 zeros
    for comb = main_comb, side_comb1, side_comb2 do
        vertexes ← comb
        img = cv2.rectangle(img, vertexes)
    end for
▷ apply add_angle for angle deviation between two side_combs
▷ apply angle for rotation of the overall shape
▷ here we only exhibit the main parameters when calling functions
    RotationMatrix = cv2.getRotationMatrix2D(center, angle)
    img = cv2.warpAffine(img, RotationMatrix)
return img
end function

for main_width, main_height, side_width1, side_width2, side_height, angle, add_angle, distort_degree do
▷ all parameters are adequately and randomly sampled within a reasonable range
    main_comb = (main_width, main_height)
    side_comb1 = (side_width1, side_height)
    side_comb2 = (side_width2, side_height)
    img = H_SHAPE_DRAWING(main_comb, side_comb1, side_comb2, angle, add_angle)
    Using the Wand package to distort the img
    img.distort(method =' arc', (distort_degree, ))
    Reshape img back after distortion
    Export and save img as 64 × 64 binary matrix
end for
```

Supplementary References

[S1] Sohn, K., Lee, H. & Yan, X. Learning structured output representation using deep conditional generative models. *Adv. Neural Inf. Process. Syst.* **28**, 3483-3491 (2015).

[S2] Kingma, D. P., Rezende, D. J., Mohamed, S. & Welling, M. Semi-supervised learning with deep generative models. *Adv. Neural Inf. Process. Syst.* **27** (2014).

[S3] Kingma, D. P. & Welling, M. Auto-encoding variational bayes. *In Proc. 2nd Int. Conf. Learning Representations* (ICLR, 2014).

[S4] Yao, Y., Rosasco, L. & Caponnetto, A. On early stopping in gradient descent learning. *Constr. Approx.* **26**, 289–315 (2007).
